# Supplementary figures and images for: Long-lived central memory γδ T cells confer protection against murine cytomegalovirus reinfection
Source: PLoS Pathog. 2024 Jul 8;20(7):e1010785. doi: 10.1371/journal.ppat.1010785 (PMC11257398; doi:10.1371/journal.ppat.1010785)

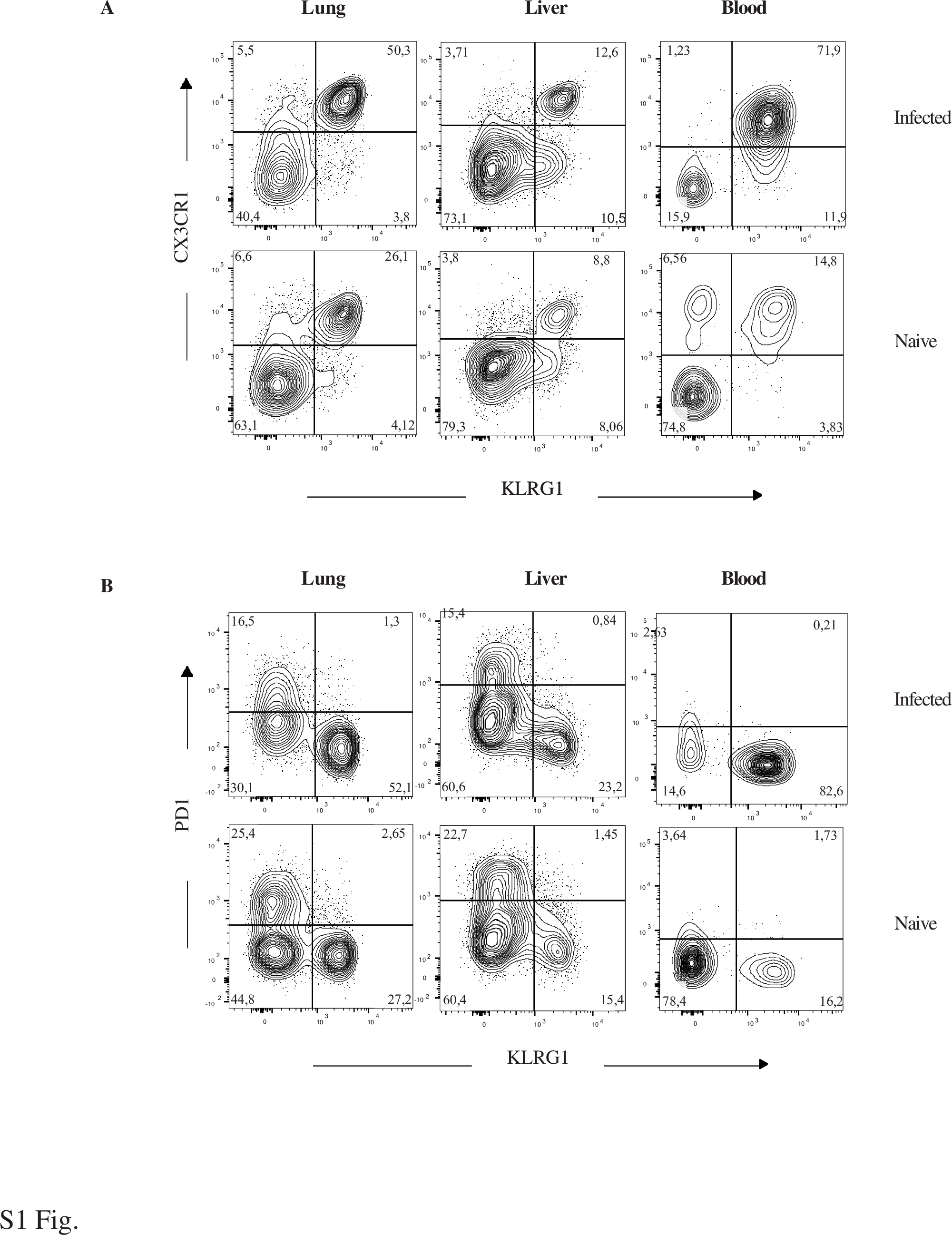

Supplement: S1 Fig — (A) Co-expression of CX3CR1 and KLRG1 on γδ TEM from organs and blood of d92 MCMV-infected and age-matched control mice. (B) Mutually exclusive expression of KLRG1 and PD1on γδ TEM from organs of d92 MCMV-infected and age-matched control mice. One representative mouse is shown for each (control and long-term MCMV infected) group of mice. (TIF) [file ppat.1010785.s001.tif]

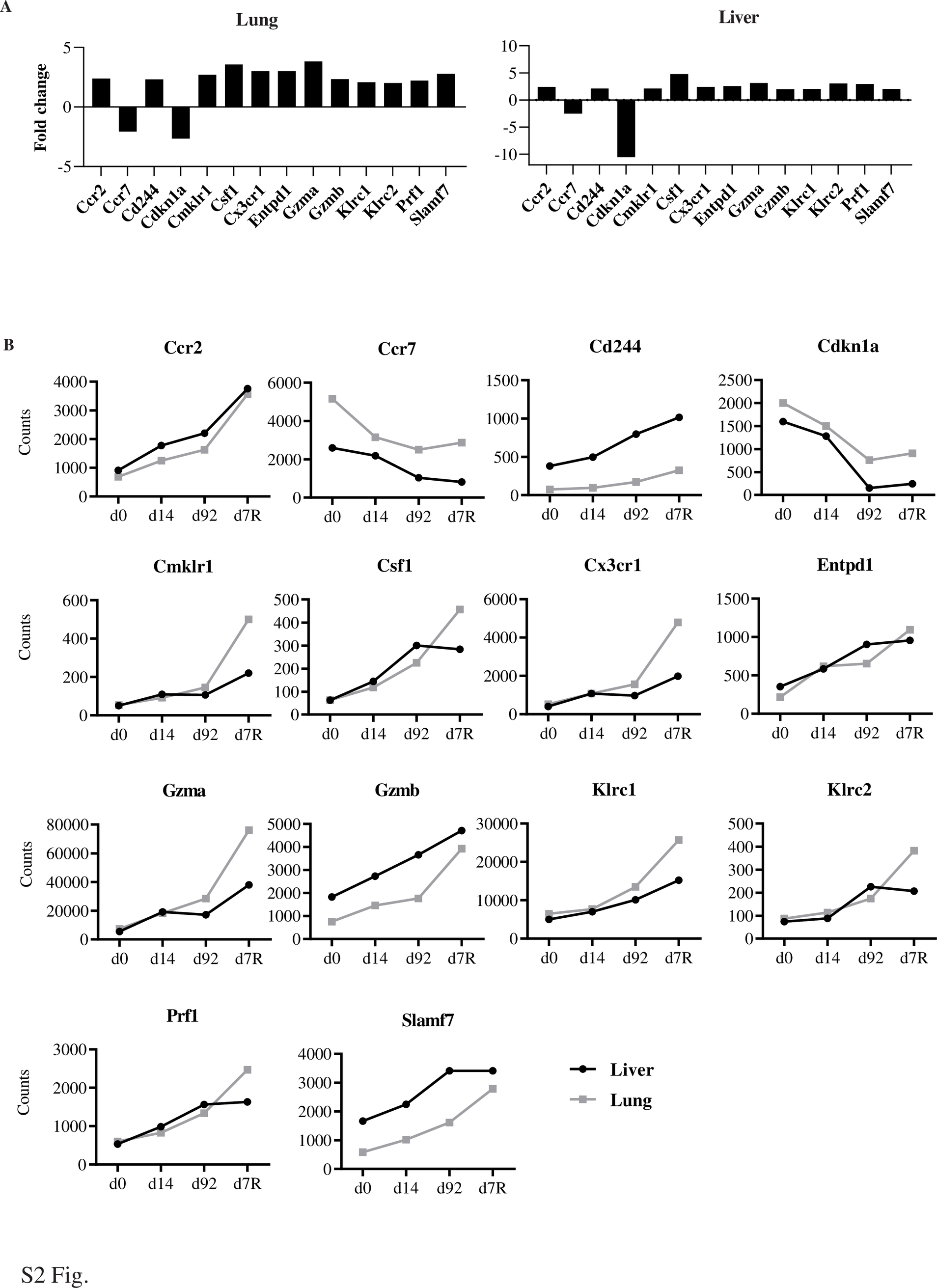

Supplement: S2 Fig — γδ T cells were sorted from lungs and liver of TCRα-/- mice (n = 10) at d0, d14 or d92 post-primary MCMV infection, at day 7 post-reinfection, and pooled before RNA extraction. Analyses were performed with nSolver Analysis Software (NanoString). (A) Histograms represent transcripts shared by liver and lungs, and whose d92/d0 ratios were >2 or < -2 (minimum counts = 100). (B) Counts evolution of theses transcripts at day 0, 14, 92 and day 7 post reinfection. (TIF) [file ppat.1010785.s002.tif]

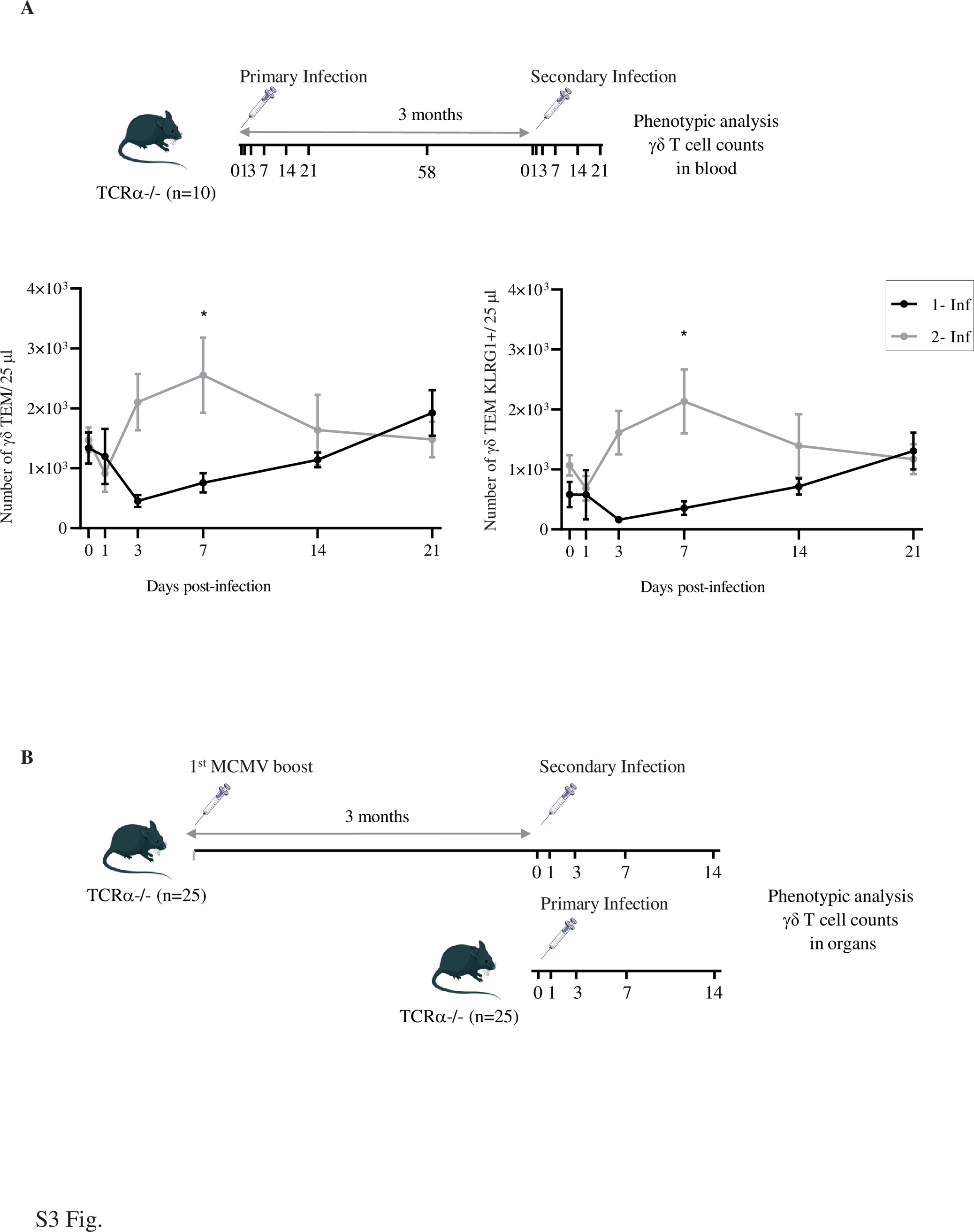

Supplement: S3 Fig — Analysis of γδ T cell memory through subsequent MCMV infections: (A, top) Experimental scheme for longitudinal analysis of γδ T cells in blood. TCRα-/- mice (n = 10) were infected with MCMV (2.103 PFU) at day 0, then re-challenged at day 92 with similar dose of MCMV. Mice were bled at day 0, 1, 3, 7, 14, 21 and 58 post-primary infection and at day 0, 1, 3, 7, 14 and 21 post-secondary infections. (A, bottom) Comparative analysis between primary (1- Inf) and secondary infection (2- Inf) of γδ TEM (left) and γδ TEM KLRG1+ (right). (B) Experimental scheme for analyses in organs. TCRα-/- mice (n = 25) were infected with MCMV (2.103 PFU) at day 0, or left uninfected (n = 25). Three months later, uninfected mice were primarily infected, and infected mice were re-challenged with similar dose of MCMV. Mice (5) were euthanized at indicated time points for phenotypic analysis and viral load quantification in the liver and lung. Images of mice and syringes were drawn by using pictures from Servier Medical Art. Servier Medical Art by Servier is licensed under a Creative Commons Attribution 3.0 Unported License (https://creativecommons.org/licenses/by/3.0/). (TIF) [file ppat.1010785.s003.tif]

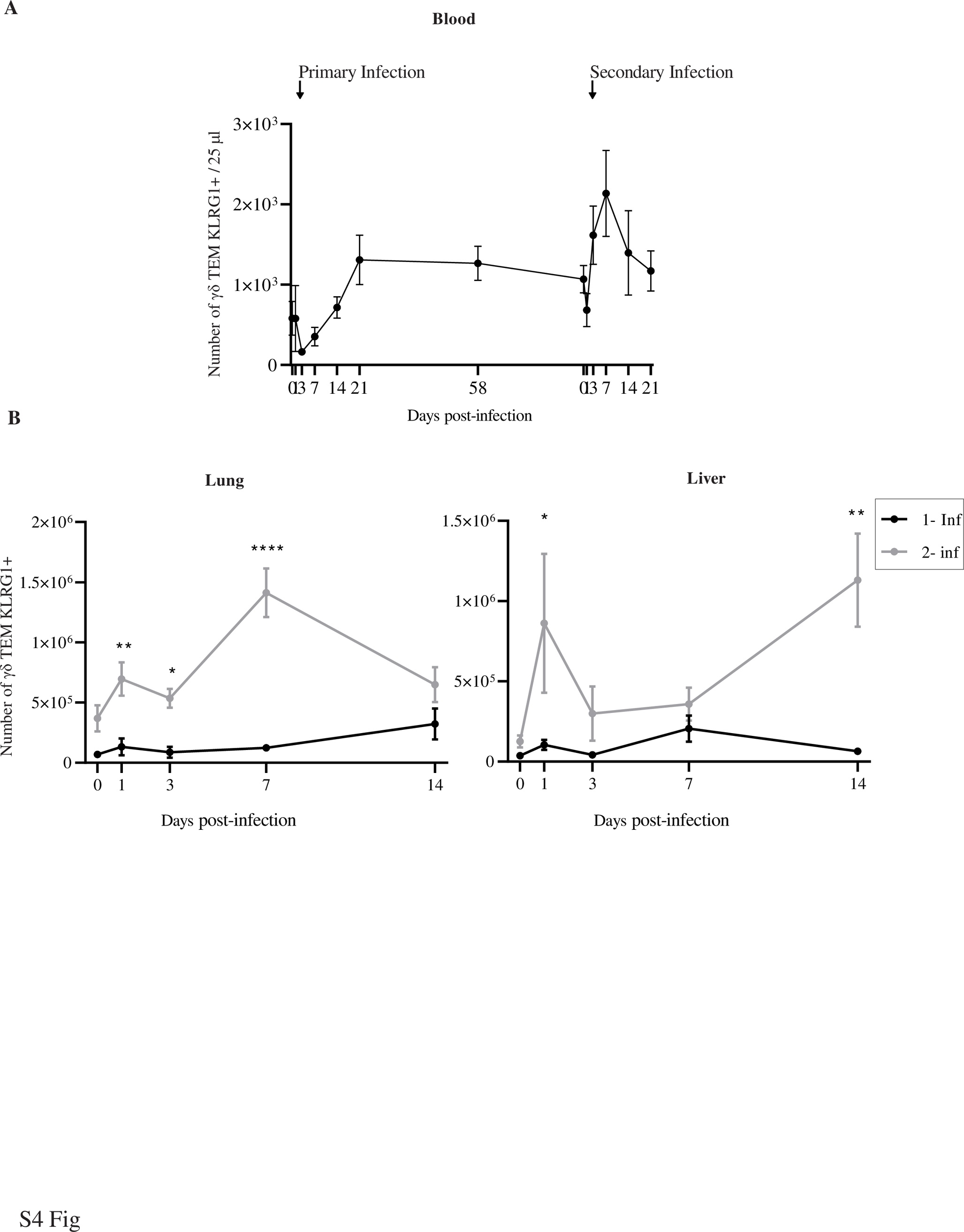

Supplement: S4 Fig — (A) Longitudinal analysis of KLRG1+ γδ T cells in blood following the experimental scheme depicted in S3A Fig. Mean absolute numbers +/- SEM of KLRG1+ γδ T lymphocytes in 25 μl of blood are shown. (B) Comparative analysis between primary (1- Inf) and secondary infection (2- Inf) of γδ TEM KLRG1+ numbers in lung and liver (2-way ANOVA). The experiment was repeated twice with concordant results. (TIF) [file ppat.1010785.s004.tif]

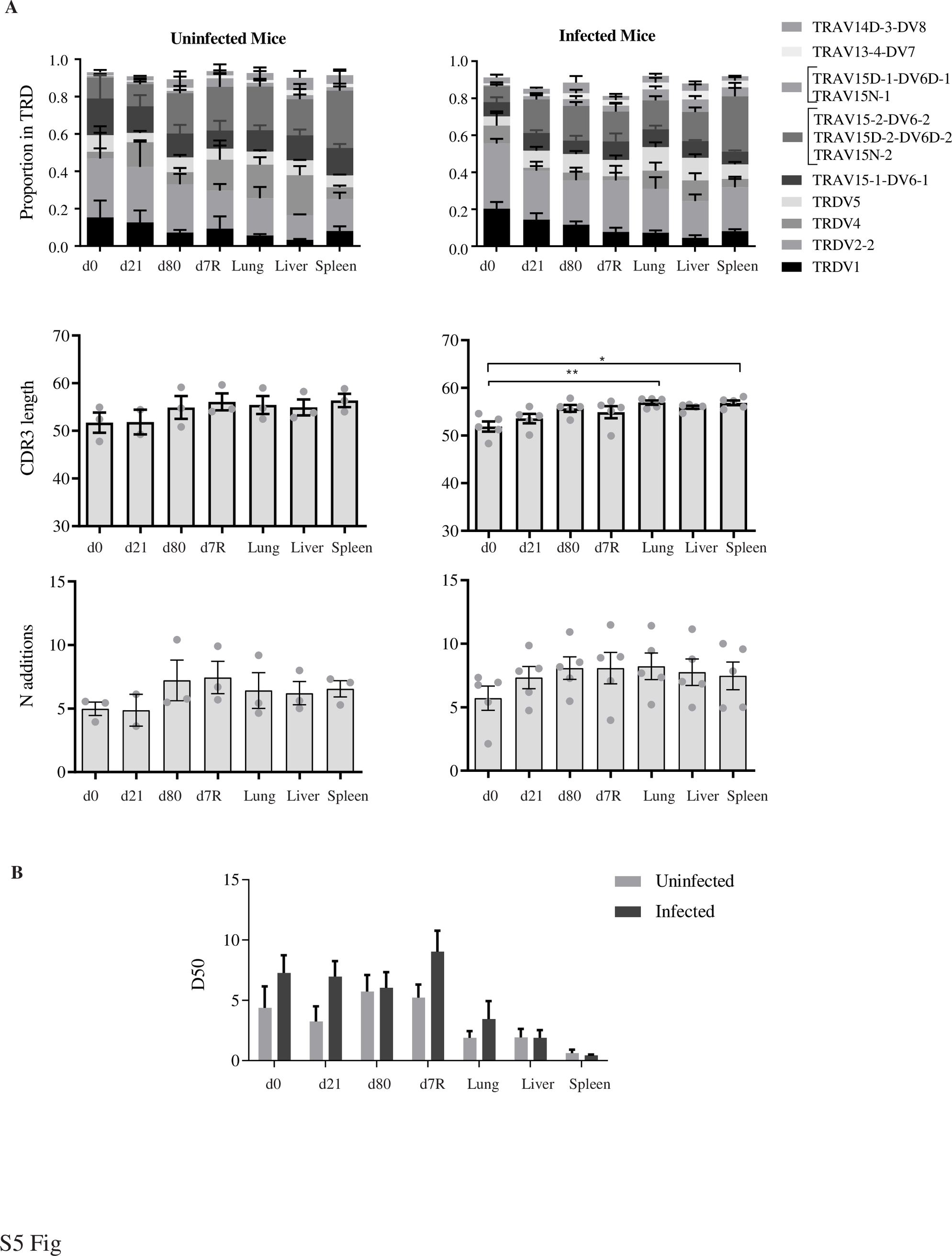

Supplement: S5 Fig — TCRα-/- mice (n = 5) were infected with MCMV. Age matched uninfected TCRα-/- mice (n = 3) injected with medium were used as controls. Blood was drawn at different time intervals post infection. At day 7 post reinfection, blood and organs were analyzed. (A) Comparison between infected (right panels) and age-matched control mice (left panels), of the CDR3 TRD repertoire of blood samples at day 0, 21, 80 post-infection (d0, d21, d80) and at day 7 post-reinfection (d7R), and from organs at d7R. (Upper panels) TRDV usage distribution, (Middle panels) CDR3 length in nucleotides (including the codons for C-start and F-end residues), each dot represents the weighted mean of an individual sample. (Lower panels). (Lower panels) Number of N additions, each dot represents the weighted mean of an individual sample. (B) Percentage of unique clonotypes required to account for 50% of the total repertoire in infected or age-matched control mice. Statistical test was 1-way ANOVA. (TIF) [file ppat.1010785.s005.tif]

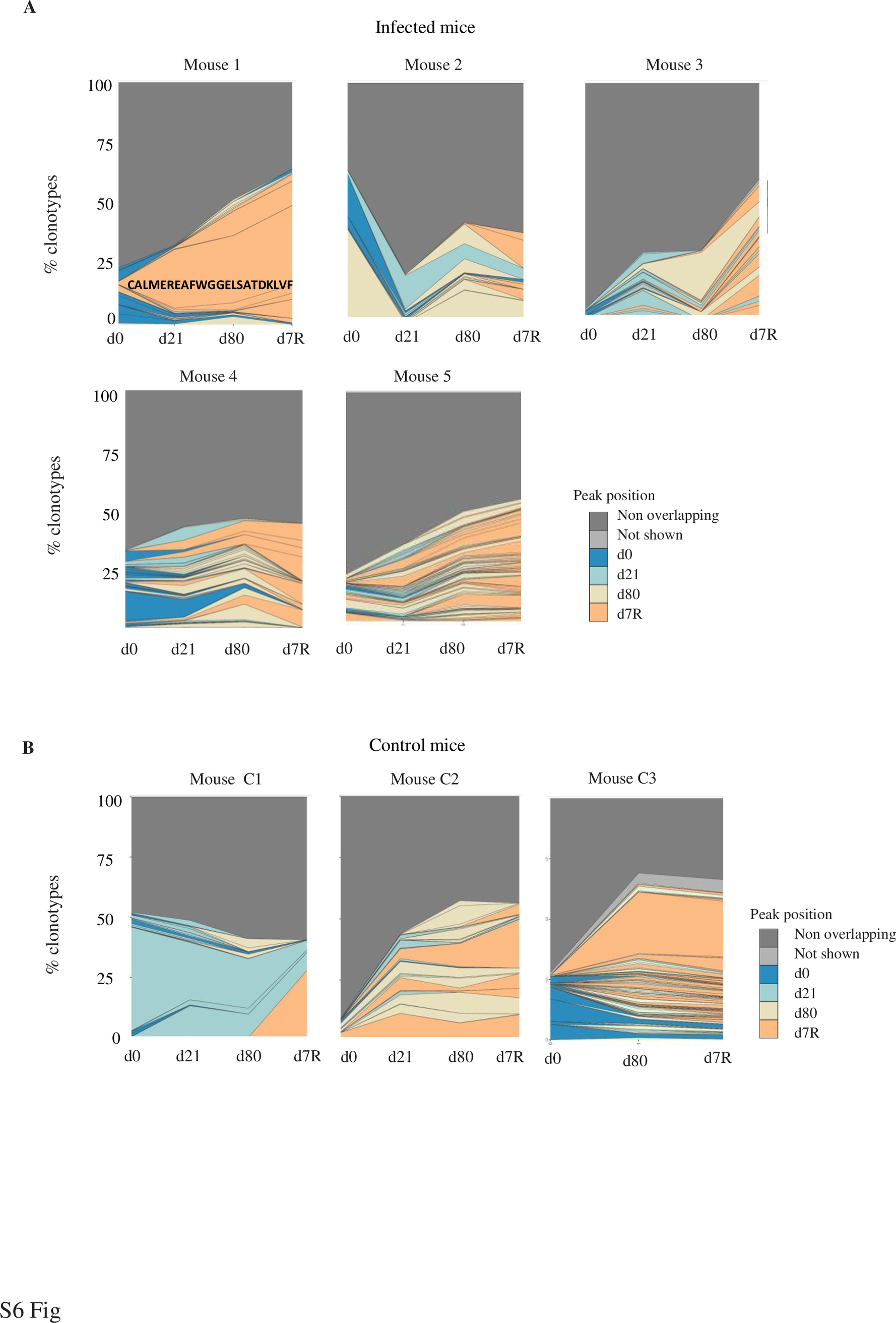

Supplement: S6 Fig — TCRα-/- mice (n = 5) were infected with MCMV. Age-matched uninfected TCRα-/- mice (n = 3) injected with medium were used as controls. Blood was drawn at different time intervals post-infection and at d7 post-reinfection. Clonotype tracking stackplots are shown with detailed profiles for top 100 clonotypes, as well as collapsed (“Not-shown” in light gray) and non-overlapping (dark gray) clonotypes found in blood of MCMV-infected mice (A) or age-matched control mice (B). Clonotypes are colored by the peak position of their abundance profile. (TIF) [file ppat.1010785.s006.tif]

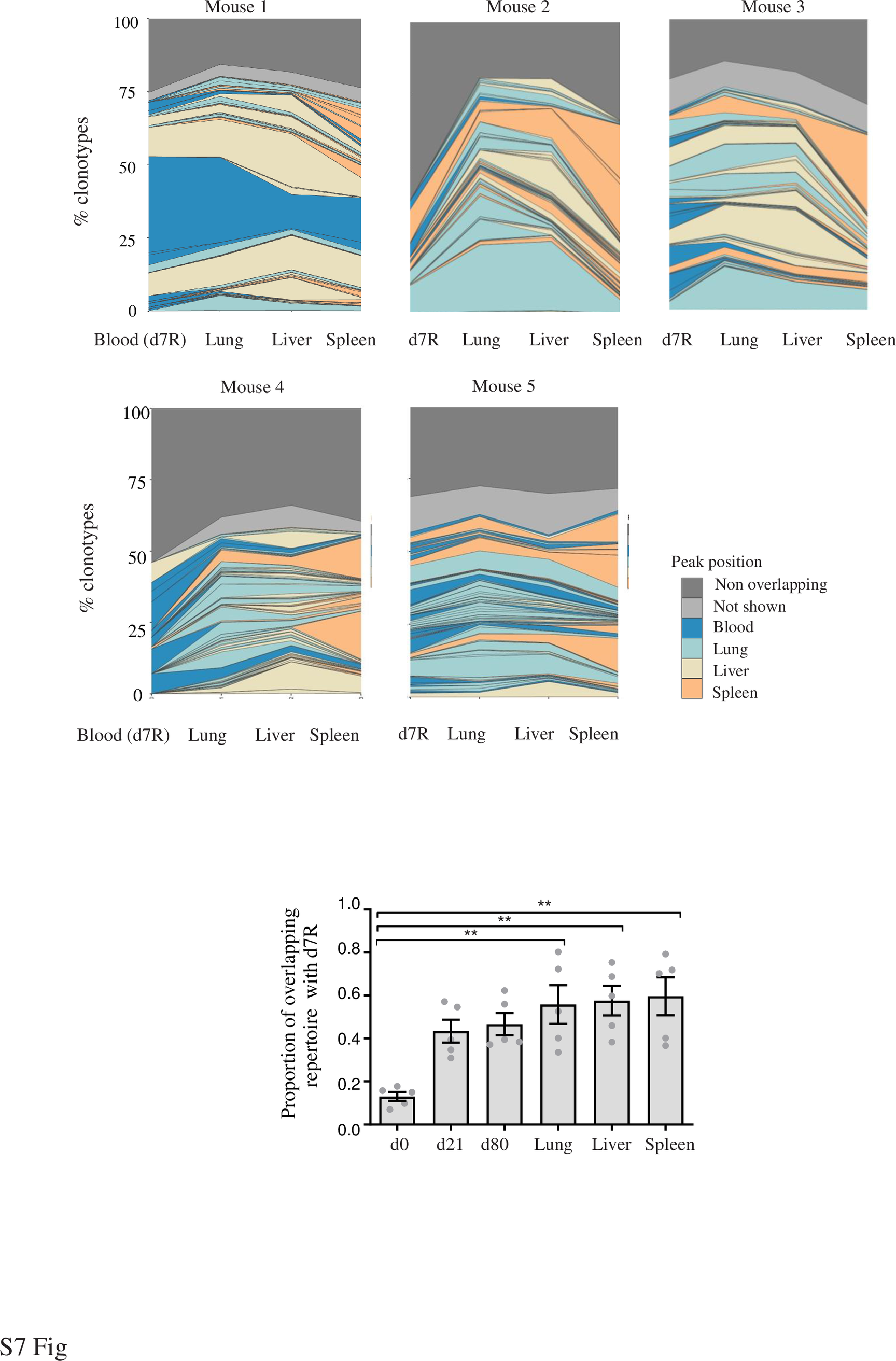

Supplement: S7 Fig — TCRα-/- mice (n = 5) were infected with MCMV and reinfected 3 months later. At day 7 post reinfection (d7R), blood and organs were analyzed. (Upper panels) Clonotype tracking stackplots for the infected mice: detailed profiles for top 100 clonotypes, as well as collapsed (“NotShown” in light gray) and non-overlapping (dark gray) clonotypes found in blood and organs at d7R. Clonotypes are colored by the peak position of their abundance profile. The colors are matched for each mouse samples but not between the different mice. (Lower panels) Overlap frequencies of the D7R repertoire compared to the indicated column. Each dot corresponds to one pair comparison and one mouse. Statistical test was 1-way ANOVA. (TIF) [file ppat.1010785.s007.tif]

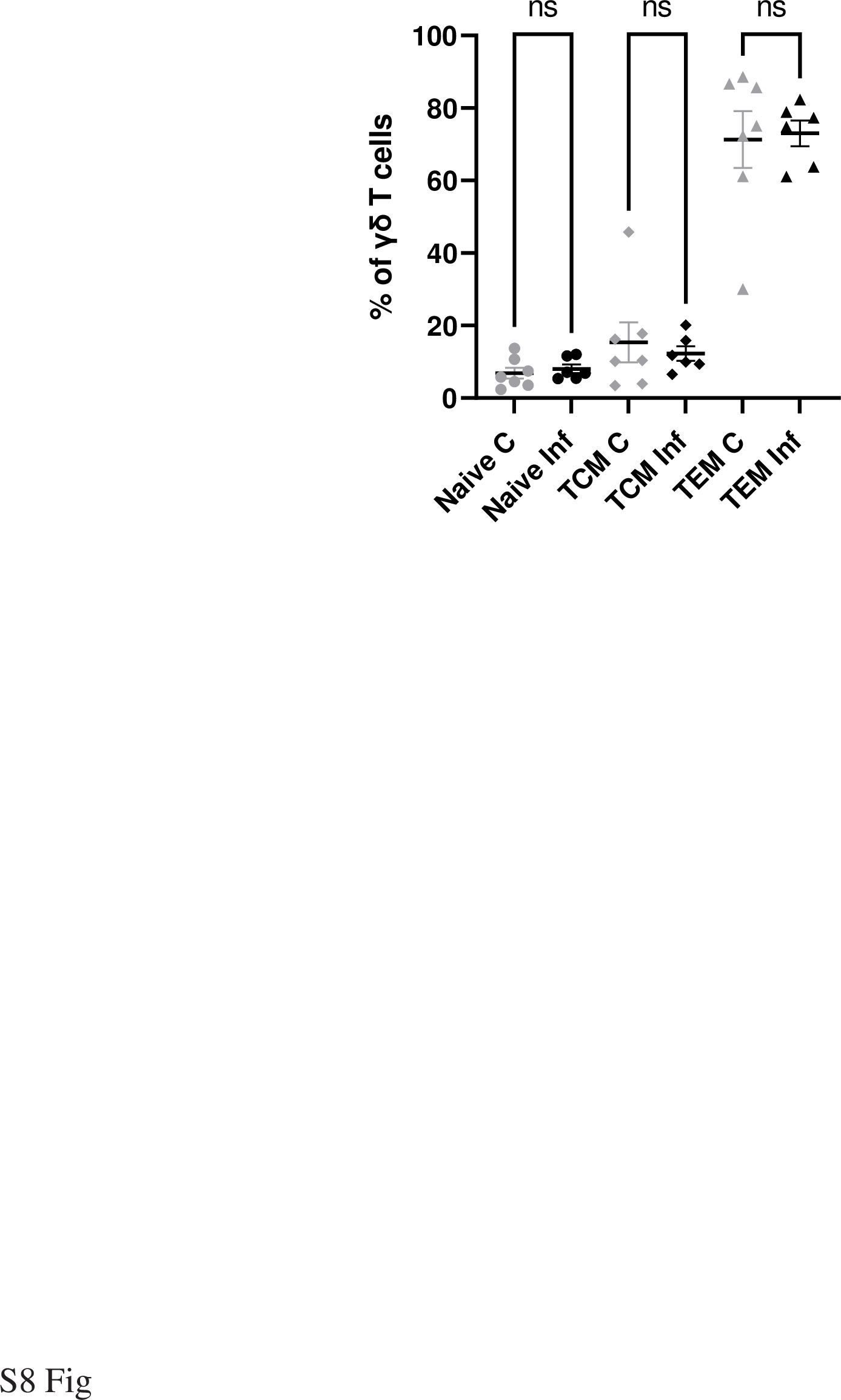

Supplement: S8 Fig — Percentages of γδ naïve (CD44-CD62L+), TCM (CD44+CD62L+) and TEM (CD44+CD62L-) in the spleen of 6 infected and 7 age-matched uninfected mice are shown, as well as the mean+/- SEM. One way ANOVA for comparison. (TIF) [file ppat.1010785.s008.tif]

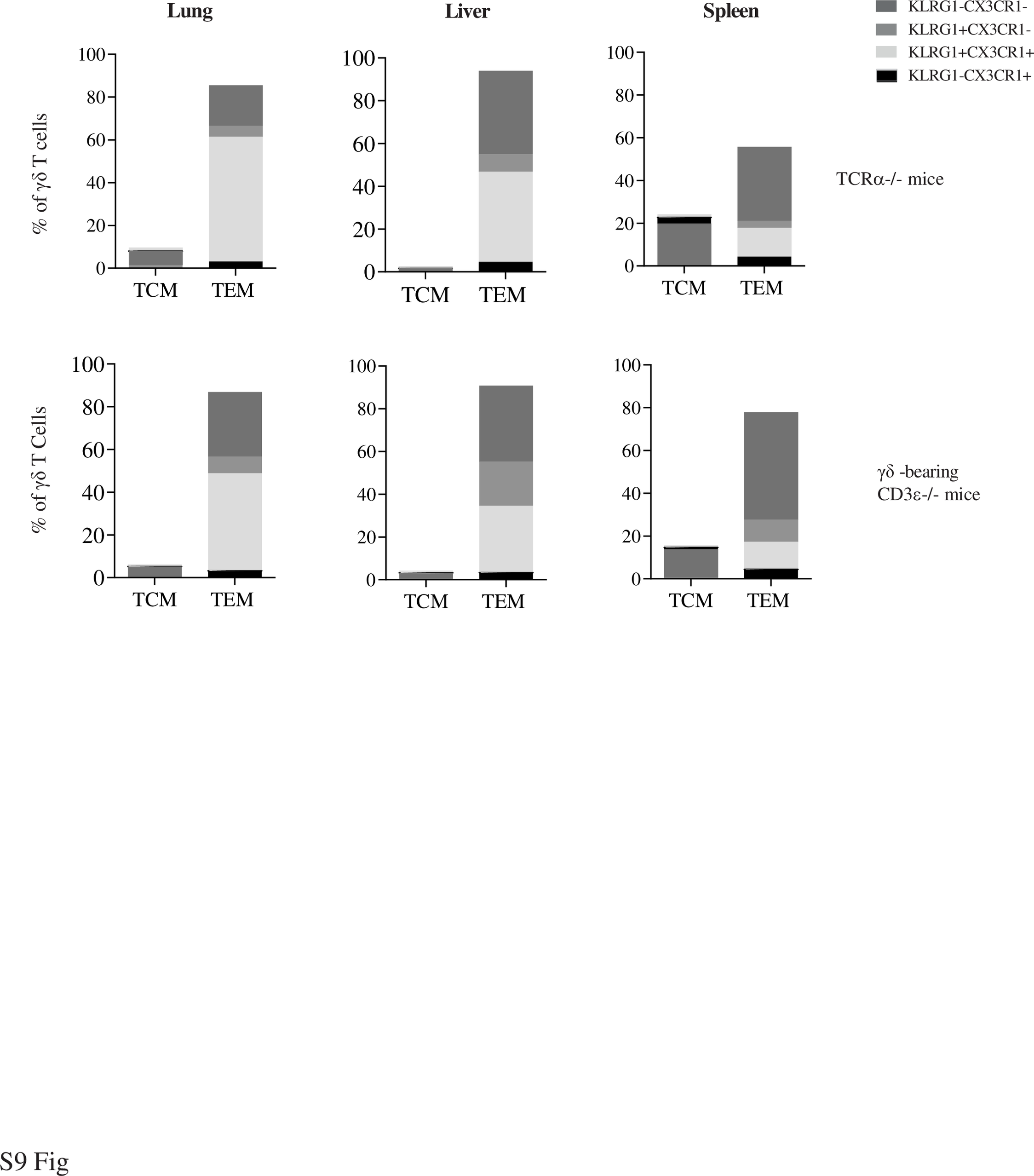

Supplement: S9 Fig — (Upper panels) Percentages of γδ TCM and TEM were determined in organs from 5 d92-MCMV infected TCRα-/- mice. (Lower panels) Long-term MCMV-induced γδ T cells were sorted from the spleen of TCRα-/- mice and transferred into CD3ɛ-/- mice that were subsequently infected with MCMV. Percentages of γδ TCM and TEM were determined in organs from 8, γδ bearing CD3ɛ-/- mice that had survived until d130. The proportion of indicated subtypes within total TCM and TEM is shown by shades of grey. (TIF) [file ppat.1010785.s009.tif]

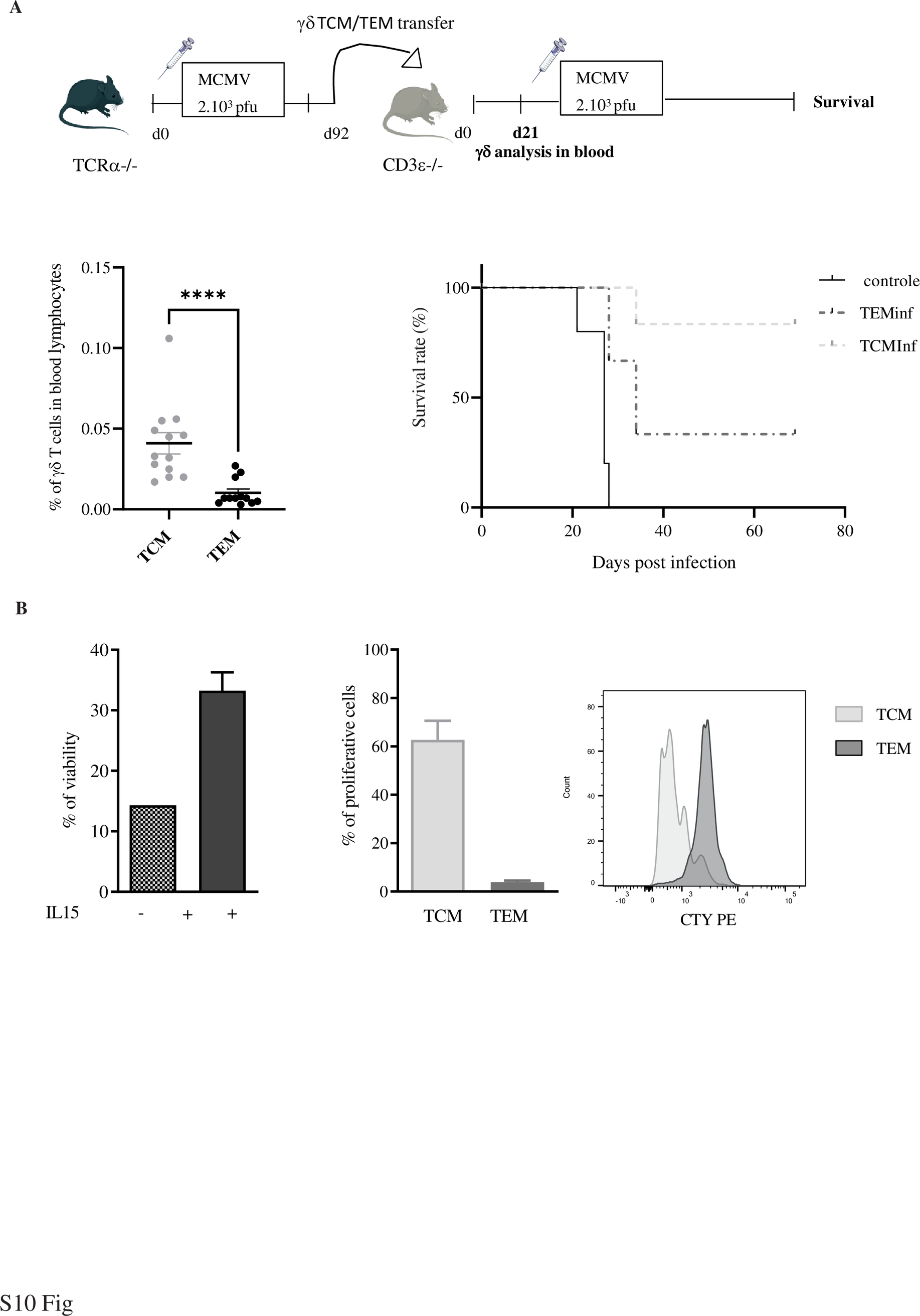

Supplement: S10 Fig — (A, top) Experimental scheme. Total γδ T cells were sorted from the spleen of 3 months MCMV-infected TCRα-/- mice. γδ TCM and TEM subsets were sorted and transferred in CD3ɛ-/- mice (200000 cells/host). (A, lower left) Data represent percentages of γδ TCM and TEM among blood lymphocytes in individual mice, 3 weeks after transfer. (A, lower right) 3 weeks after transfer, mice were infected with MCMV concomitantly to control CD3ɛ-/- mice (2.103 PFU). Two experiments were performed with concordant results and were pooled in the figure. Horizontal bars show the means +/- SEM (Mann Witney) (B) Splenocytes from 3 months infected mice were labelled with CTY and cultured with or without IL-15 (200 ng/μl) for 3 days. (B, right) Percentages of viable cells among γδ T cells cultured in the absence or presence of IL-15. (B, middle) Percentages of proliferative γδ TCM or TEM after 3 days of culture with IL-15 (B, right) Representative histograms of cellular proliferation for TCM (light grey) and TEM (dark grey) from d92-infected TCRα-/- mice. Data are representative of 3 independent experiments. Images of mice and syringes were drawn by using pictures from Servier Medical Art. Servier Medical Art by Servier is licensed under a Creative Commons Attribution 3.0 Unported License (https://creativecommons.org/licenses/by/3.0/). (TIF) [file ppat.1010785.s010.tif]

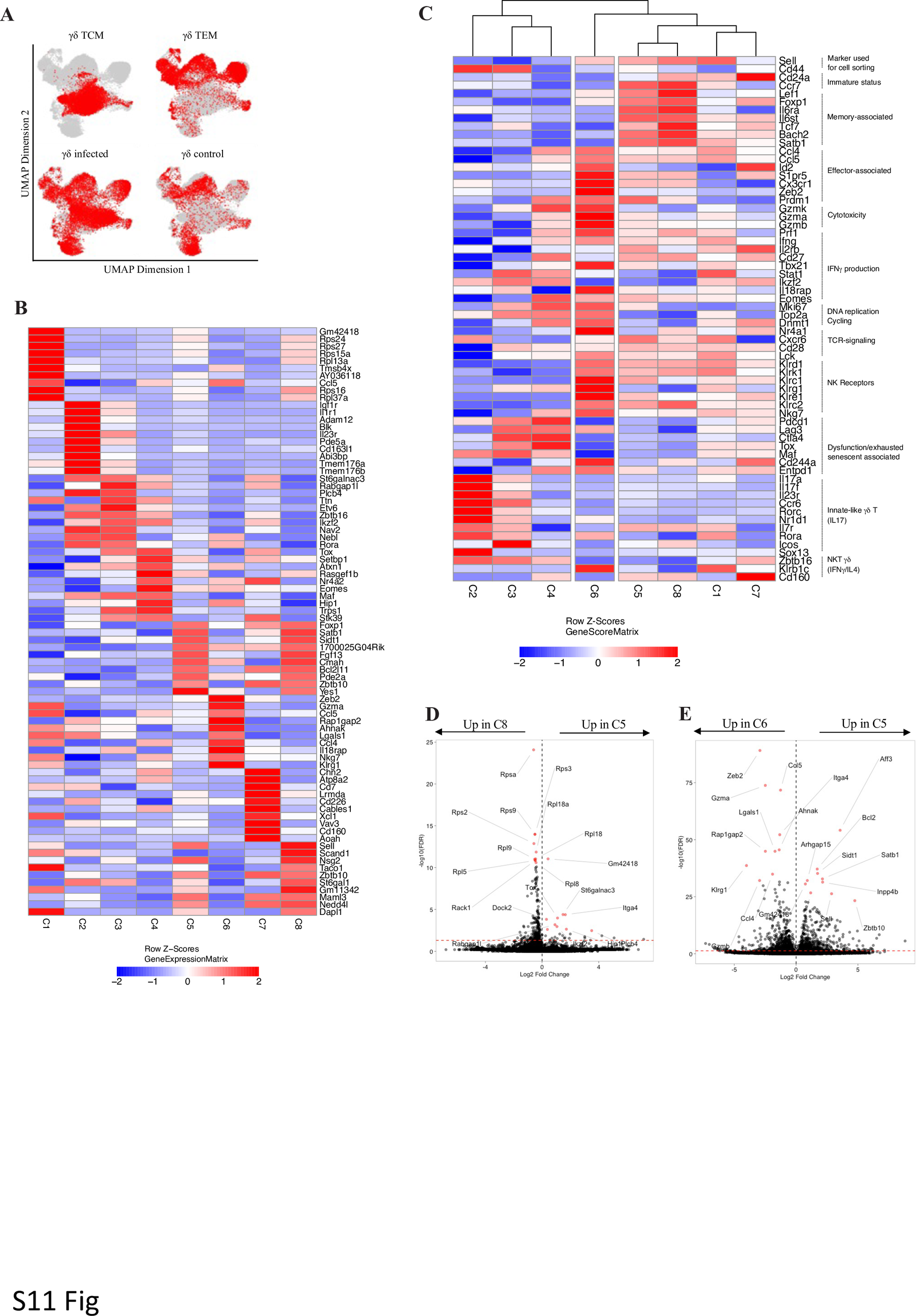

Supplement: S11 Fig — (A) UMAP projection of sample-originated cells. Cells from each sample were highlighted in red on the same UMAP embedding. UMAP algorithm was performed on the combined LSI scATAC-seq and scRNA-seq as described in Mat & Meth section. (B) Heatmap illustrating the top 10 up-regulated differentially expressed genes within clusters C1 to C8. Values are represented using the scaled of the mean log-normalized gene expression across cells among clusters (column). Row Z-scores are constrained to an upper bound of 2 and a lower bound of -2. (C) The heatmap shows the scaled of the mean log-normalized gene score matrix (prediction of a gene expression based on the accessibility) across cells among clusters (column) for a list of selected genes (row), categorized by ’functionality’. Column clustering was performed using Euclidean distance and the Ward.D2 method. Row Z-scores are constrained to an upper bound of 2 and a lower bound of -2. (D) Volcano plot representing the top 10 of up- and down-regulated genes (labeled red dots) between C5 and C8, based on the gene expression matrix. The dashed red line indicates the FDR ≤ 0.05. (E) Volcano plot representing the top 10 of up- and down-regulated genes (labeled red dots) between C5 and C6, based on the gene expression matrix. The dashed red line indicates the FDR ≤ 0.05. (TIF) [file ppat.1010785.s011.tif]
