# Supplementary material for: Long-lived central memory γδ T cells confer protection against murine cytomegalovirus reinfection
Source: PLoS Pathog. 2024 Jul 8;20(7):e1010785. doi: 10.1371/journal.ppat.1010785 (PMC11257398; doi:10.1371/journal.ppat.1010785)
Supplement: Attachment — Submitted filename: Response to editor and reviewers.pdf [file ppat.1010785.s021.pdf]

Response to editor and reviewers

We answered to specific reviewer's questions below in the point by point reply.

Regarding the general question from the editor about the differences between TCM and TEM on their ability to respond after resting and to retain proliferative functions, we performed two types of additional experiments:

1- *We adoptively transferred TCM or TEM gd T cells from three months infected mice and waited three weeks before infection of the recipients mice. S10A Fig. depicts the maintenance of gd TCM in the absence of antigen, while few gd TEM were found in the blood, showing a better survival capacity of TCM. Consistently, only recipient mice adoptively transferred with gd TCM 3 weeks earlier survived MCMV-infection in this novel setting. These results are now included in the manuscript (lines 333-339 and S10 Fig.).*

   In an attempt to compare gd TCM and TEM proliferation in vivo, we sacrificed some mice five days after infection of recipients and analyzed Ki67 expression in memory gd T cell subtypes in MCMV-target organs. We did not obtain statistical differences regarding the proportion of Ki67+ cells among gd T cells in infected recipient mice bearing TCM vs TEM (see figure below):

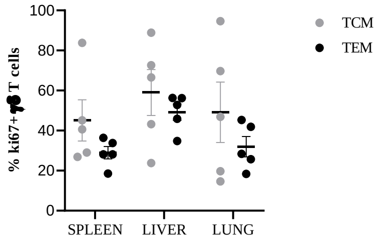

   This result might be explained by the fact that Ki67 stains cells that have proliferated and not only cells that are proliferating. In contrast, we obtained clear results in vitro showing higher proliferative potential of gd TCM vs TEM in the presence of IL15 (S10B Fig). In vivo the better control by TCM than TEM could also be explained by the better survival of TCM when compared to TEM.

2- *We made a scRNAseq analysis combined to ATACseq analysis to compare the transcriptome and epigenome of either gd T cells from the spleen of uninfected versus infected mice, or gd TCM versus TEM from the spleen of infected mice. As presented in Fig 9, clear transcriptomic and epigenomic differences were evidenced between gd T cells from MCMV-primed and unprimed gd T cells, as well as between the two memory subsets. Sorted TCM and TEM from infected mice distributed in completely different clusters. Among differences, we found increased accessibility and transcription of bcl2 in gd TCM, in agreement with their enhanced survival potential. By contrast, TEM overexpressed CD40-ligand which is consistent with effector functions. Moreover, this analysis revealed a transcriptomic and epigenetic signature specific of infection in gd TCM, leading to the hypothesis that gd T cell memory is the consequence of intrinsic molecular changes after MCMV encounter.*

Responses to Reviewer's Questions

Reviewer #1:

- The statistical analysis in Fig.1B is not clear. What kind of post-hoc test was used for multiple comparisons, and what reference was used? In Fig.1C, day 3 was used as reference - why? There is no statistical analysis of the MCMV copy data- Data presentation in general: the authors may want to consider showing an overlay of primary and secondary responses as line or bar diagram to make comparisons between different responses easier, rather than showing a continuous line throughout primary and secondary responses.

A suggested by the reviewer, we re-formatted the figures showing the kinetic of gd T cell counts on an overlay of primary and secondary responses (upper panels Fig 4B, S3A Fig and S4B Fig), and we used a 2 way ANOVA test for multiple comparisons. The text was changed accordingly to the novels figures (lines 213-215).

Regarding MCMV copy data, since MCMV copies were detected 3 months post-infection (baseline for re-infection), in contrast to day 0 (baseline for primary infection), we now presented the data of viral load evolution as fold changes from each baseline (lower panels Fig 4B), and we used 2-way ANOVA for comparison. The text was changed accordingly to the novels (lines 219-223)

- Fig.1B and Fig.2: the authors should comment on why there is such a sharp peak on day 1 in the secondary response in the liver that then declines by days 3-7. Any theory?

The liver is the first organ affected after intraperitoneal injection of MCMV. A rapid gd T cell response may occur locally. The following decrease could be due to rapid colonization of other organs through the circulation of KLRG1+ cells and/or to death of local gd T cell responders.

- Fig.S1: there are only relatively minor changes in the constitution of the gd compartment during the secondary infection. Doesn't this lack of clear functional changes within the gd population reflect a lack of a bona fide memory response, other than an expansion?

The important increase of gd TEM percentages in three months infected mice, with 80% of TEM in lung, liver and blood (old S1 Fig and novel Fig 1A) makes it difficult to have substantial changes of TCM/TEM composition during secondary infection. Nonetheless, the novel representation - suggested by this reviewer with overlay of primary and secondary responses - allow to better see these changes during secondary infection. In addition, these phenotypic changes are confirmed by functional changes as observed in our Nanostring analysis, with the increased gene expression of cytotoxic markers at 7 days post-reinfection (S2B Fig).

- Fig.5B: it's surprising to see the authors completely dismiss the effect of FTY720 treatment in the liver when the p value is at 0.055. It could be argued that the experiment was simply underpowered and that its design prevented this p value from falling below 0.05. A statement like "no statistical differences were evidenced" (lines 229-230) is simply not *good enough to discuss the potential biological effect of FTY720 treatment.*

We thank the reviewer for this interesting comment. We totally agree with that and we changed the text in the novel version of the manuscript as follows: Lines 191-192 *"Thus, as for αβ T cells, FTY720 primarily affects lymph nodes egress and re-circulation of gd TCM. After treatment, viral loads were slightly increased in organs from d92-infected mice, near to statistical significance (Fig 3B)."*

- Fig.7A: this figure should show data from all mice not just a selection of some.

We added the requested missing mice and created a new figure (S6 Fig)

Reviewer #2:

- Figure 1. For some individuals no virus could be detected (I guess 1 means between 0 and 1). Did the authors find any correlation between negativity/low virus load and any of the other parameters? The authors should address and discuss this, even if it might complicate the statistical analysis (also for the following figures).

We thank the reviewer for this interesting comment. We assessed whether differences in viral loads were associated with the levels of gd TEM in organs but did not find any correlation.

- Figure 5. The data are clear and underline the role of tissue-resident cells. Did the authors or others also perform similar experiments for αβ T cells or wild type mice? If yes, it would be interesting to learn about it in the manuscript.

We reorganized the manuscript for sake of clarity, now starting with the analysis of gd T cells in three months infected mice. The results from Fig 5 are now depicted in Fig 3 in the new version of the manuscript. We only did this experiment in TCRa-/- mice. The group of Christopher Snyder (Philadelphia, PA) did similar experiments in wild-type mice in order to understand the maintenance of the inflationary ab CD8 T cell pool. The effect of FTY-treated mice only affected naïve ab T lymphocytes subset while no significant changes in the numbers of inflationary ab CD8 T cells (which were mostly CD44+CD62L-) were observed in the blood, liver and lung. They concluded that maintenance of MCMV-specific effector T cells does not depend on migration through or antigen recognition within the lymph nodes (Smith et al. 2014. PLoS Pathog. 10: e1004233).This reference is now added in the discussion (Lines 406-409).

- Figure 6. The authors treat animals with the γδ TCR-modulating antibody GL3 and find a clear reduction in virus control in treated mice. They interpret this as evidence for a role of the TCR in virus control. Although the data are suggestive, this interpretation goes too far. Without providing further evidence, one must assume that the antibody merely binds to γδ T cells and that a general impairment of γδ T cell function will be caused by antibody-induced TCR modulation. Therefore, this experiment provides no evidence for a direct role of the γδ TCR in virus control. It is just a very nice confirmation of the results obtained with the deficient mice and the adoptive transfer experiments.

We agree with the reviewer that control experiments checking for gd T cell functional capacities after GL3 treatment was missing in the first version of the manuscript. We now performed a control experiment by testing the capacity of gd T cells isolated from TCRa-/- mice treated for one week with GL3 to produce IFNg upon activation with PMA and ionomycin. As shown in Fig 5B, GL3 treatment did not affect the capacity of gd T cells to respond to activation when compared to gd T cells from isotypic control mAb treated mice. This result strengthen our conclusion that gd TCR plays an important role in the control of viral load.

- Figure 7. The authors should explain the significance of an overlapping TCR-repertoire. Actually, I find the entire part difficult to understand. I also found the remark quite interesting that injection

of medium caused also some effects. The authors may consider and discuss that changes in the repertoire reflect a type of stress response of the "adaptive/adaptate" gd T cells and not a specific response to MCMV. In such a setting the TCRs would serve as a kind of bar code of expanded clones rather than as indicators of an antigen-specific clonal expansion. Actually, one of the corresponding authors (JDM) mentioned this concept in a recent editorial (PMID: 33245813) discussing (PMID: 32981055).

The term overlapping repertoire is referring to identical gd TCR sequences retrieved between different mice (public sequences, shared between two mice). The results are expressed as the percentage of overlapping repertoire between each different mice (pairwise comparison). The fact that this overlap markedly decreases in blood and organ gd T cells after secondary challenge indicates that the repertoire is more private after reinfection.

Manipulation of mice and injection of medium can indeed induce a stress in mice that could affect gd T cells. Some of the gd T cells responding to this type of stress may be part of the response to MCMV. However, the difference in overlapping TCR-repertoire mentioned above highlights a difference between MCMV secondary infection and second injection of control medium which could reflect a different response to unspecific stress or to MCMV.

We do not fully agree with this reviewer that we should expect a public response to CMV. The public response to CMV has only been observed in congenital human CMV and relies on TCRs that most probably rearrange during fetal life. The repertoire of Vd1 gd T cells (the subset responding to CMV) in human adults has been shown to be highly private, even in CMV-seropositive individuals that show a restriction of Vd1 T cell repertoire (PMID: 28248310).

- Figure 8 and 9. I think these figures are the most important for demonstrating memory formation (irrelevant whether antigen-specific or not). It clearly demonstrates that splenic γδ T cells from infected animals protect better than cells from uninfected animals and that TCM are more potent than TEM. The authors should provide numbers/frequencies of splenic TCM of naïve and infected animals. This would give the reader an idea to which extent the lacking protection by transferred naïve cells is due to a lower overall frequency of TCM or to a qualitative change within the TCM (or both).

We compared the percentages of TCM/TEM in the spleen of same age naïve and long-term infected mice. As shown in S8 Fig and lines 306-307 (the new version of manuscript), no major differences were observed suggesting that the increased antiviral potential of MCMV-primed gd T cells is not due to higher numbers of gd TCM.

Other points

- **Sometimes the term "important(ly)" is used when changes in frequency and numbers are described (lines 171,225,253,284). This is already an interpretation. It would be better to use terms like clearly, strongly, pronounced or the like.**

As suggested by reviewer #2, the term important(ly) is now changed and underlined in the new version of manuscript.

Reviewer #3:

- **Are the authors concerned that MCMV goes latent? Is there reactivation during the 3 months between virus injections?**

As suggested by the reviewer, MCMV is indeed a virus that maintains in a latent form in wild type mice. The situation is much less clear regarding TCRa-/- mice that we are using in this study. In fact there are no data available on MCMV latency in TCRa-/- mice. It is plausible that as for wild-type mice, MCMV can be found in TCRa-/- mice in both replicative and latent states depending on the cell type, and that replication in these immunodeficient mice is more frequent than in wild type mice. The lower immune pressure due to the absence of ab T cells may give a selective advantage for the virus to replicate. As a matter of fact, viral loads in organs from TCRa-/- mice were found to be generally higher than in wild-type mice (Khairallah, Plos Path 2015). As discussed in the present manuscript, we were still able to detect DNA copies of MCMV in organs three months post-infection using a qPCR method, but we did not look specifically for the presence of replicative versus latent MCMV. However, independently of the viral status, we are able to ask in this model whether a novel MCMV boost would induce faster/higher gd T cell recall response. This is also because of the limit of this successive infection model that we conducted adoptive transfer experiments.

- In Fig 1-2, are the cells measured during the secondary infection the same cells that have seen virus the first time? Or new cells responding? Or cells that have been primed during latency?

As stated in the manuscript the results of the TCRd repertoire analysis suggest that the secondary gd T cell response is composed by clones primed during primary infection (represented in light blue in supplemental Fig 6) as well as many new responding clones (represented in orange in supplemental Fig 6). The figure below focuses, giving 2 infected mice as examples, on the few clones that show the typical expected adaptive-like response to MCMV i.e. that expand at day 21 when compared to d0 and that re-expand between d80 and d7R.

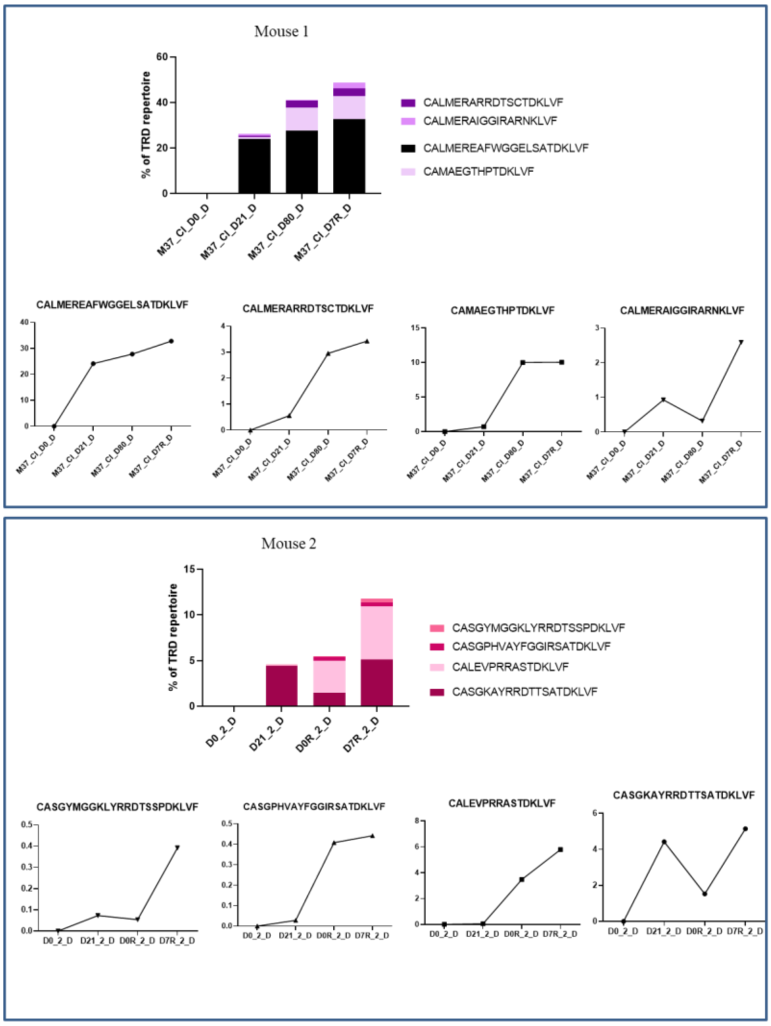

Example with clonotypes that had frequencies at D7R>D80/D21 and D21/D80>D0 for 2 infected mice

However, when we looked globally to the TCR repertoire, we observed no increase in repertoire clonality (S5B Fig) indicating that there is not a focalization of the repertoire on specific clones already selected during primary infection and that even if they expand faster, the clones that respond during re-infection are not necessarily those that expanded during the primary response.

This notwithstanding, we also used a new approach to cluster TCR sequences according to the similarity of their CDR3. This method is based on the observation that in alpha/beta T cells, CDR3aa differing by only one amino acid, can recognize the same peptide. Using this type of analysis we observed a significant increase in mean cluster size for d21/d7R compared with d0/d7R. This increase was not observed in uninfected mice (New Fig6C). This closer amino acid sequences of CDR3aa between d21 and d7R versus between d0 and d7R only in infected mice, suggested a shared $\gamma\delta$TCR response against CMV antigens during primary and secondary infection.

Regarding putative priming during latency, Fig S7 comparing the D7R with D0, D21 and D80 indicates that the biggest change in TCRd repertoire is seen between D0 and D21, while D21 is similar to D80. This suggests that the cells were primed at the first infection rather than during latency (if we consider that latency may occur during the period between D21-D80).

- How is it that the authors are able to challenge the same mice with MCMV twice? Are there no neutralizing antibodies against MCMV?

The absence of ab CD4 T cells in TCRa-/- mice most likely affects the production of neutralizing antibodies against MCMV although it might be interesting to test in another study whether gd T cells can have a regulatory role in that context.

- Regarding the enhanced clearance of the virus observed in Fig 1B, can the authors attribute this to gd T cells? Or is it simply neutralizing Abs, or even memory NK cells (which have previously been proposed in the setting of CMV infection)?

CD3e-/- mice dye from MCMV infection (see for instance Fig7B and C) showing that NK cells cannot afford long-term protection in the absence of T cells. Since memory NK cells have been described, and that CD3e-/- mice dye relatively late post-infection (~ 50 days post-infection, Fig 7), it is possible that the virus mutates to escape NK cell control in CD3e-/- mice. In TCRa-/- mice however, it is likely that innate memory participate to viral clearance after secondary infection. However, the substantial increase (about 2 log) of viral loads in GL3 mAb versus isotype-treated mice 7 days post-reinfection proved the important role of gd T cells on viral control (Fig 5)

- The antibody blocking experiments should probably go into supplement, as I'm not sure how to interpret the findings. Plus, a key control that is missing is gd TCR blockade in naïve mice infected with MCMV. Would the authors see equally high titers in this setting as well?

By contrast to reviewer's comment, we think that the anti-gd TCR blocking experiment is very important in our demonstration so we would like to keep these results as a main figure. As interestingly suggested by reviewer, we tested gd TCR blockade during primary infection (results shown in Fig 5B). Importantly, the effect of anti-gd TCR injection in primarily infected mice was delayed (day 21) comparatively to secondary infection (day 7) demonstrating once again a more rapid response of gd T cells during secondary infection.

- The authors should determine the antigen-specificity of the gd T cells. This may help explain why the gd T cells contract and not inflate like some T cell populations during MCMV.

By contrast to ab T cells, gd T cells do not recognize peptides presented by MHC-I molecules. They are believed to recognize stress-induced self antigens but very few of them have been identified so far, particularly in mice. This represent a main challenge in the field of gamma-delta T cells, and even if it is with no doubt an interesting question, it is an issue beyond the scope of this paper.

- If there is clonal expansion, I'm confused why there is no enrichment of specific clones following secondary infection (in fig 7B).

This question is answered in the previous question by this reviewer.

Other points

- It is a bit awkward to re-show the fig 1B-C data in Fig 2A.

We agree with the reviewers and decided to show only the data of naïve TCM and TEM subsets and to remove the figure on total gd T cells (old Figure 1).

- In Fig 3, the actual flow plots showing CX3CR1 vs KLRG1 rather than graphs would be clearer.

We do not really understand this comments since flow plots are shown in Fig S1 (old Fig S2).
