# Supplementary material for: Long-lived central memory γδ T cells confer protection against murine cytomegalovirus reinfection
Source: PLoS Pathog. 2024 Jul 8;20(7):e1010785. doi: 10.1371/journal.ppat.1010785 (PMC11257398; doi:10.1371/journal.ppat.1010785)
Supplement: Attachment — Submitted filename: Response to reviewers Yared et al. 2024.pdf [file ppat.1010785.s022.pdf]

Responses to reviewers

Reviewer #2: In general, I am fine with the answers to my previous comments and the revision. Just two points.

Fig S2B. Please enlarge the symbols or give them different colors.

As requested we enlarged Fig S2B symbols and used two different colors for lung and liver.

Fig. 5 The authors may discuss.

Fig. 5 B and D. Despite that gd T cells of the antibody treated animals can be activated in a TCR-independent manner (PMA/Iono), TCR-antibody might affect effector function/survival of the primed/trained cells triggered by innate/cytokine receptors.

Fig. 5D gdT cells may be primed/trained in a TCR independent manner (first two weeks) and but primed/trained cells require TCR-mediated signals to execute effector functions in the third week (Fig. 5D). This would be in line with a MCMV TCR-specific response. Alternatively, the TCR antibody could provide a TCR-mediated signal interfering with effector functions triggered by innate/cytokine receptors (see above).

We agree with the reviewer and added the following sentence in the discussion (p21): "Our TCRγδ-blocking experiment suggest that TCR-signaling is important for viral load control after reinfection. However, we cannot fully exclude that γδ TCR-antibody also affects effector function or survival of innate or cytokine receptor-dependent activation of γδ T cells".

Reviewer #4: The manuscript by Yared et al. describes an interesting adaptive-like feature of gd T cells in response to MCMV infection, extending on two previous publications appearing almost at the same time. In their manuscript, the authors elaborate on the crucial question whether immunological memory is generated in the gd T cell compartment. One of the previous publications already suggested a major difference in protecting immunodeficient mice when gd T cells from infected mice were used in transfers. In the current manuscript, the authors then start to characterize the features and functions of the memory-like gd T cells.

The major problem in the characterization is the assumption that well-established markers for memory subpopulations of CD8+ aß T cells, particularly TEM and TCM are transferable to the gd lineage. Without comparing the respective populations side by side, this is a dangerous misconception, in my opinion, as it paves the way for future simplifications in following studies.

Characterization of γδ T cell memory subsets based on previous knowledge on αß T cells has been useful in the past since it allowed, to some extent, to follow pathogen-responding γδ T lymphocytes. Indeed, their peculiar mode of Ag recognition and the paucity of knowledge on γδ TCR ligands preclude the use of tetramers.

Though, we are aware of existing differences between the two T cell subsets and we apologize since we did not want to stress that γδ TCM and TEM are strikingly similar to their αß T cell counterparts. We now added this sentence at the beginning of the results section: "Based on previous knowledge on αß T cells, we used CD44 and CD62L expression to characterize γδ TCM-like (CD44+CD62L+) and TEM-like (CD44+CD62L-) subsets, that will be further on referred to as γδ TCM and TEM for simplification".

As mentioned by the reviewer, concomitant analyses should be performed to resolve this issue that cannot be done in TCRα-/- mice. Actually, few studies analyzed concomitantly CD8 $\alpha\beta$ and $\gamma\delta$ T cells. B Lucas and B Martin defined adaptive-like $\gamma\delta$ T cells subdivided into naive $CD44^-$ and memory $CD44^+$ subtypes, as for CD8 $\alpha\beta$. These cells are mostly CD27+ and prone to IFNγ production (Lombes et al. J Immunol 2015, DOI: 10.4049/jimmunol.1500375). The transition from a naïve $CD44^-CD62L^+$ to an activated $CD44^+CD62L^-$ phenotype was observed on Phycoerythrin (PE)-responding $\gamma\delta$ T cells after immunization (Zeng et al. Immunity, 2012, doi.org/10.1016/j.immuni.2012.06.011). More recently, the group of Pabst used CD62L and CD44 and identified subsets of $\gamma\delta$ T cells with distinct turnover rates and circulation kinetics that closely mirrored migratory subsets of αβ $CD8^+$ T cells (Ugur et al., Sci Rep, 2018, 12;8(1):8986. doi: 10.1038/s41598-018-27339-8).

In addition, we have previously compared CMV-specific CD8 αβ T cells and $\gamma\delta$ T cells responding to human CMV and showed that they share a common effector memory phenotype and common expansion kinetics during the course of infection (Couzi et al., J Infect Dis. 2009 Nov 1;200(9):1415-24. doi: 10.1086/644509).

The authors already show in the experiment for Figure 1, for example, that gd cells do not compare to the classical categories, also not regarding expansion and contraction, at least to a much lesser extent. "TEM" cells would express CX3CR1 and KLRG1, in naïve mice most of them don´t, however.

As depicted in our previous analysis (Khairallah et al., PLoS Pathog. 2015 Mar; 11(3): e1004702. doi.org/10.1371/journal.ppat.1004702), the $\gamma\delta$ T cell-response to primary MCMV- infection shows a rather « classical » (non inflationary) kinetics with expansion of $\gamma\delta$ T cell numbers in organs at d7-14 followed by contraction at d21. In the long-term, $\gamma\delta$ T cells acquire a similar CD44+CD62L-KLRG1+ phenotype as so-called inflationary CD8 αβ T lymphocytes. As reported for CD8 αβ T cells, the initiation and amplitude of the $\gamma\delta$ T cell response, as well as the proportion of CD44+CD62L-KLRG1+ cells in the long-term might depend on the viral dose (Redeker et al. Front. Immunol., 2018, 8: 1953, doi.org/10.3389/fimmu.2017.01953) . In the latter study, analysis in blood of uninfected mice showed that about 42% of CD62L- CD8 αβ T cells were KLRG1-. However, in this study and others, CD44+CD62L-KLRG1+ CD8 αβ T cells are defined as TEM, while we only used CD44 and CD62L. This emphasizes the importance of marker selection for cell-subset definition, as mentioned by reviewer.

A detailed comparison by the transcriptional landscapes would be helpful to dissect the gd subpopulation and compare them to the CD8 aß subpopulation. The analysis in sFig.2 goes in this direction. As this analysis does not differentiate the subpopulations, it is not able to clarify this critical issue, however.

This transcriptional landscape comparison between $\gamma\delta$ TEM and $\gamma\delta$ TCM has been done through single cell analysis. We thank the reviewer for this idea to compare $\gamma\delta$ to CD8 αβ subpopulations. However, as mentioned above, we think that to be informative these comparisons should be done using $\gamma\delta$ and CD8 αβ T cells from the same CMV-infected mice. Since we are using TCR α -/- mice this is unfortunately not possible

Strangely, a significant attempt to differentiate the subpopulations is made in Figure 9, most likely because of the previous review process. These "new" data should be shown and discussed after Figure 1 and include a much more detailed bioinformatic analysis, possibly including a comparison with CD8 aß TEM and TCM as they can easily be obtained from deposited data sets.

As suggested by reviewer, a new version of our single cell analysis is now presented (figure 9), and a novel analysis is discussed in the results section. We think that these new data are interesting to describe after our transfer experiment, showing a differential protective function of splenic $\gamma\delta$ TCM-like and TEM-like cells, while Fig 1 displays lung and liver.

These new data have been confined to the GEO repository under the accession number GSE269394. To review GEO accession GSE269394: Go to https://www.ncbi.nlm.nih.gov/geo/query/acc.cgi?acc=GSE269394. Enter token oxknemsuppuznwh into the box.

Reviewer #4: **The analysis in Fig. 2 is important and the data appear clear in showing that most tissue-based gd T cells 3m after infection appear in the blood. It would be important to strengthen this finding by a control experiment for tissue-resident gd T cells in the same experimental setup, even from the same mice. Dermal or intestinal gd T cells would be very good controls.**

As mentioned in the result section (p9), we used a well-validated labeling technique of circulating cell by intravenous injection of conjugated CD45 mAb (Anderson et al. Nat Protoc 9, 209–222 (2014) doi: 10.1038/nprot.2014.005). As a control experiment, when setting up the experiment shown in Fig 2, we compared flushing or not with buffer, the lung and liver vasculature, for few mice. The proportion of stained CD45+ cells was highly reduced (80%) in the lung and, to a lesser extent (60%) in the liver, sustaining the removal of circulating cells only upon perfusion.

In Fig.3 the strong reduction of TCM in lung, liver and blood after FTY720 treatment is interpreted as block of lymph node egress. This statement is not valid unless data are shown that TCM are, in fact, residing and, after treatment, accumulating in lymph nodes. They might be otherwise affected.

FTY720 has been regularly used as a blocker of T cell egress from lymph node. In the setting of MCMV infection, FTY was used to show that maintenance of MCMV-specific effector CD8 T cells does not depend on migration through or antigen recognition within the lymph (Smith et al. 2014. PLoS Pathog. 10: e1004233).

In contrast to the strong FTY720-mediated decrease of $\gamma\delta$ TCM observed in liver, lung and blood (Fig 3), $\gamma\delta$ TCM remained unaffected in the lymph nodes (below), although we could not show an accumulation. We only dissected mesenteric lymph nodes that might only partially reflect what happens in the whole mice. So, in agreement with reviewer's comment, we amended our interpretation in the text and now mention FTY720 only as a product that allows to decrease the number of $\gamma\delta$ TCM in organs (p9).

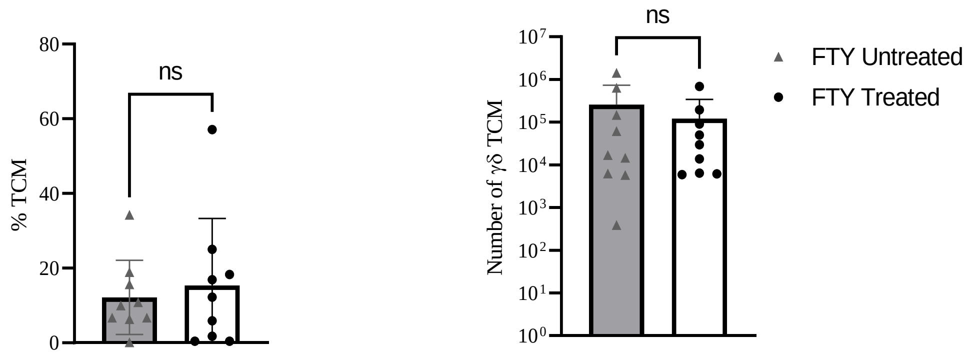

***Fig.** Analysis of the proportion (left) and number (right) of $\gamma\delta$ TCM in lymph nodes of FTY-treated and control TCRα-/- mice.*

The data in Fig. 3b should not be shown here as there is no clear conclusion possible.

In fact we did a mistake on the p values in the submitted version since the statistics where done on a single experiment whereas the data are pooled from 2 experiments. We corrected this in Fig 3B where a statistical increase (p= 0.022) is now observed in the liver, while the p value in lung is 0,188. Please find down below the raw data of viral load copy numbers where 1= limit of viral loads detection/PCR

	Lung			Liver	
Mice	FTY Untreated	FTY Treated	Mice	FTY Untreated	FTY Treated
1	3252	7339	1	4,01155	10133
2	33	2	2	54,27388	4020
3	7	15	3	7,850016	1193
4	16	2320	4	45,15332	319
5	2	39	5	2,54608	5
6	11	64	6	1	320
7	1	207	7	62,01746	96
8	105	1642	8	45,75728	184
9	56	19478	9	43,55762	14
10		1	10		5

Figure 4 shows a somewhat faster and to some extend stronger response in secondary infections. The extent is, however, much less pronounced when compared to CD8 ab responses. This should be mentioned somewhere in the manuscript.

Thanks for this comment. Since we did not analyze CD8 αβ T cells in these experiments it is difficult to know whether in this precise setting CD8 αβ T cells would have more pronounced response when compared to $\gamma\delta$ T cells. Moreover, in most analysis of CD8 T cell responses to MCMV, experiments are done using specific peptides and focusing on MCMV peptide-specific CD8 αβ T cells, which is barely comparable to the response of a whole population of $\gamma\delta$ T cells in which we do not now the proportion of cells responding to the virus. This is now mentioned in the discussion (page 18).

The changes in viral load, i.e. the potentially better protective function in the "memory" situation are not very convincing and the effect of expanding NK cells as well as the potential role of antibodies preclude a solid conclusion at the current state of the manuscript.

We agree with the reviewer that the absence of increased viral load despite re-infection presented in Fig 4B, is less convincing to show that $\gamma\delta$ T cells are important for long-term control of MCMV, than the results obtained using the anti-$\gamma\delta$ TCR mAb and presented in Fig 5. We then decided to delete Fig 4B.

In Figure 5 the effects of anti-GL3 treatment are clear regarding control of viral load. The interpretation that specific recognition by the gdTCR cannot be drawn by this experiment. The persisting gd T cells after GL3 treatment might just be severely impaired in their function. The control experiment showing IFNg production after 3h ex vivo stimulation is not sufficient. How about other effector molecules like granzymes and perforin?

We agree with the reviewer that these experiments with the GL3 mAb mainly show that gd T cells are involved in the control of long-term MCMV infection but specific recognition by the gdTCR cannot be drawn by this experiment. The anti-TCRgd mAb might have affected gd T cell functions others than IFNg production, but the extent of functions potentially involved in viral control (cytotoxicity, repair factor production, G or GM-CSF production, chemokine production, etc…) that could be tested would be too large. Thus, we tempered our conclusion of the involvement of a specific recognition by gd TCR by changing the text on page 11.

The positive effects of transfer of gd T cells from long-term infected mice is extending previous findings described in PMID: 25658831 This should at least be mentioned and the additional gain of insight should be worked out.

We agree with the reviewer that adoptive transfer with gd T cells from short-term (between 2 and 3 weeks) infected mice has been performed in (Khairallah et al, PLoS Pathog. 2015 Mar 6;11(3):e1004702. doi: 10.1371/journal.ppat.1004702 and Sell et al, PLoS Pathog. 2015 Feb 6;11(2):e1004481. doi: 10.1371/journal.ppat.1004481). However, in the present paper we infected the donor mice for three months. As shown in this figure for the reviewer, the phenotype of gd T cells is very different between mice infected for 7 days or 3 months. This difference is also shown in nanostring analysis (sFig 2). Moreover, viral load after a week of GL3 treatment if not affected in mice infected for 14 days by contrast to mice infected for 3 months (Fig 5C and D). So gd T cells are clearly different between short-term and long-term infection, and it was important to assess their protective function upon adoptive transfer. This gain of insight is now specified in the text (p13).

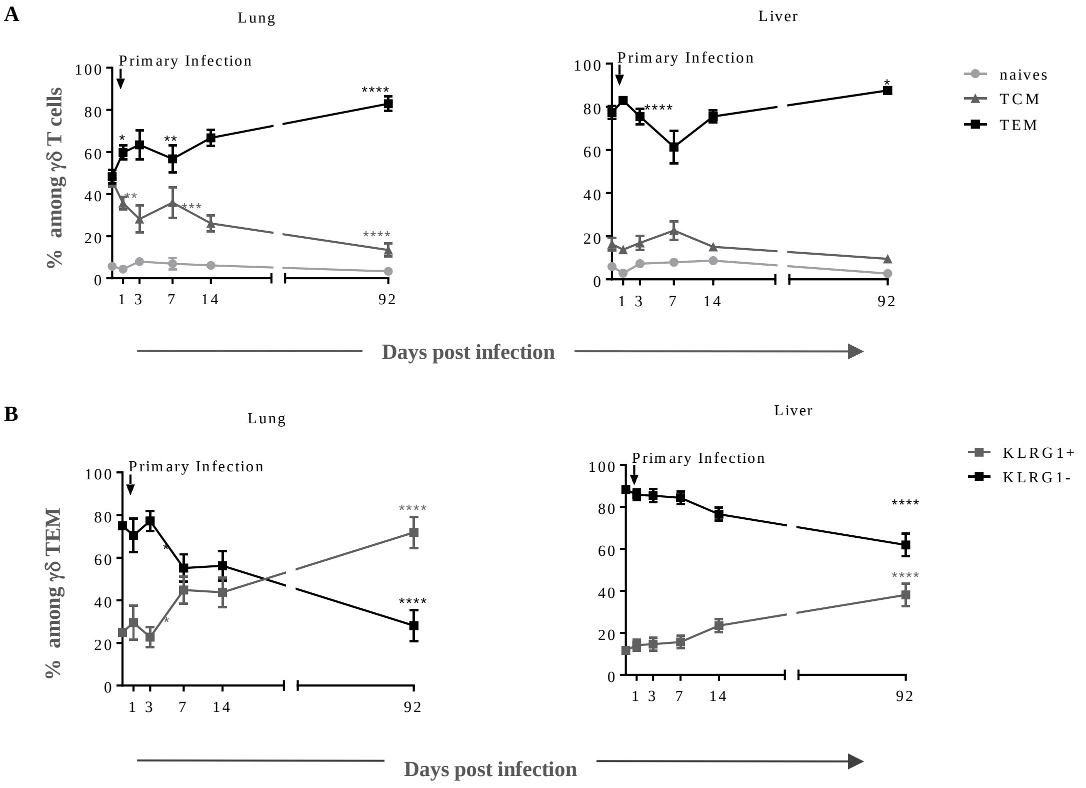

Fig. A. *Evolution of the proportion of naïve, TCM and TEM $\gamma\delta$ T cells in organs along the course of MCMV infection in TCRα-/- mice.* ***B****. Evolution of the proportion of KLRG1+ cells among $\gamma\delta$ TEM.*

Figure 8 and 9 are the most crucial parts in the manuscript as a clear protective role of TCM-like cells is shown by adoptive transfer and analyzed in detail by the scRNA experiment. The weakness is, however, that here only splenic gd T cell populations are used for analysis as well as transfer. Given the potential importance of virus persistence / latency in lung and liver, only "TEM-like cells" from the respective organs might confer protection upon transfer. This would lead to a fundamentally different conclusion, in my opinion.

Transfer experiments using γδ T cells from liver and, above all, from lung are more difficult due to poor γδ T cell numbers. However, we performed some experiments and, in the case of liver, we were able to sort KLRG1+ and KLRG1- TEM subsets. As shown in the figure below, no protection was evidenced using either subset.

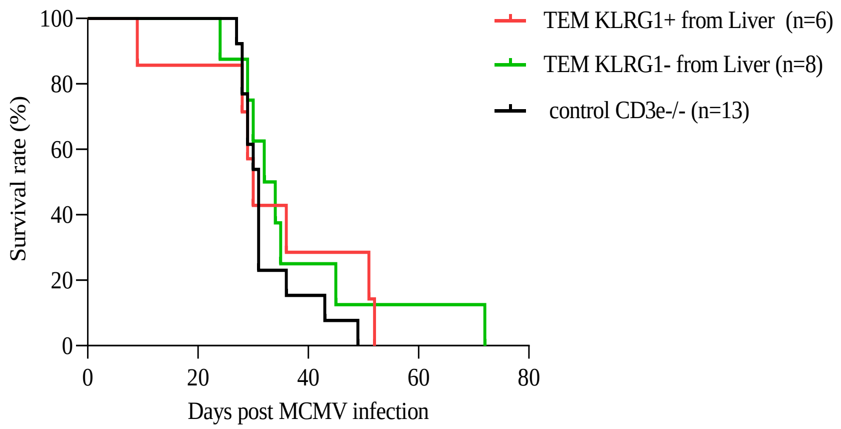

Fig. *Survival curve of CD3ε-/- mice receiving 200 000 KLRG1+ and KLRG1- γδ TEM from liver of three months infected TCRα-/- mice. The number of mice for each transfer group is indicated.*

Figure 9 needs more attention in the text, and the analysis appears rather rudimentary. At least a description of the 8 UMAP clusters should be presented, for example by displaying the top up- or downregulated genes and signature genes as bubble or violin plots

We changed the novel text and figures (Fig 9 and S11 Fig). Top 10 genes for each cluster are shown in S11B Fig.

Minor issues: The y-axis in Fig. 5 c and d should be labeled MCMV copies / ug DNA

Thanks to reviewer for it attentive reading. We have changed this label.

Reviewer #5: **I had a chance to review the article "Long-lived central memory γδ T cells confer protection against murine cytomegalovirus reinfection" by the group of M. Capone that was resubmitted to PLOS Pathogens upon in initial review. Since one of the primary reviewers appears to be unavailable, I was asked to step in during the secondary review. In fairness to the authors, my review does not address questions that were not raised by reviewers during primary review, but in fairness to the process, I had to go through a detailed assessment of the article and raise objections where I identified concerns about data and their interpretation. Overall, I think that the study is interesting and relevant to the readers of PLOS Path., but that several item need to be addressed as major concerns that require additional experimental evidence.**

The list below is in order of appearance in the text:

1. In Fig. 3B, the authors were requested address the weak stat power of their experiment, resulting in a borderline p value. They addressed this request by altering the interpretation of results, which is not an appropriate course of action: they should instead repeat the assay, generate additional evidence and define if the FTY720 effects on viral loads are significant and reproducible or not.

In fact we did a mistake on the p values in the submitted version since the statistics where done on a single experiment whereas the data are pooled from 2 experiments. We corrected this in Fig 3B where a statistical increase (p= 0.022) is now observed in the liver, while the p value in lung is 0,188. Please find down below the raw data of viral load copy numbers where 1= limit of viral loads detection/PCR

	Lung			Liver	
Mice	FTY Untreated	FTY Treated	Mice	FTY Untreated	FTY Treated
1	3252	7339	1	4,01155	10133
2	33	2	2	54,27388	4020
3	7	15	3	7,850016	1193
4	16	2320	4	45,15332	319
5	2	39	5	2,54608	5
6	11	64	6	1	320
7	1	207	7	62,01746	96
8	105	1642	8	45,75728	184
9	56	19478	9	43,55762	14
10		1	10		5

Viral loads in organs of treated or untreated mice were quantified by qPCR. Values represents MCMV copy numbers in 1 µg of total DNA.

2. In Figure 4B, the authors show viral loads as fold changes relative to Day(0) upon primary or secondary infection. They start from a value of 1 in either scenario to show a drastic increase in primary, but not in secondary infection. The problem is that at day 0, the mice were MCMV naïve and the value had to be 0. Therefore, any increase would end up as an infinite-fold increase. The authors have obviously used an arbitrary value for the day 0 of the primary infection, which is not immediately obvious. This needs to be explained more accurately.

Thanks for this comment. The arbitrary value that has been used for day 0 of naïve mice is the detection limit of the PCR used to titrate the virus. However, since these viral load results did not convince several reviewers, we decided to delete them from this new version of the manuscript.

3. In reference to the first comment by Rev#3, the authors have indeed produced no data that the MCMV indeed establishes latency, rather than persisting in their system. Since it is well known that CD4 T cells are required for MCMV control in salivary glands, it is reasonable to assume that their model may be affected by MCMV persistence in this organ (and spurious dissemination of virus to other organs from this focus of persistence). At a minimum, the authors need to perform plaque assays on SG homogenates at a late time post infection. Ideally, they should perform it on all organs.

The conclusions of this assay will not preclude publication, but they will substantially influence data interpretation (e.g. they may indicate that there is no additional spike in viral loads upon reinfection, because the virus is lytically persistent even before reinfection). Hence, this evidence is critically important.

We agree with the reviewer that our model is not a model of virus latency (as explained in our previous response to reviewer #3) and sorry if there is a misunderstanding because we do not want to claim that this is the case. We know from our viral load data that we can detect up to 10^3 DNA viral copies in organs three months after infection (see e.g. Fig 3B and Fig 5C). This maintenance of viral load is probably due to the absence αβ ab T cells in this model, and more particularly CD4 T cells (Jonjic et al. J Exp Med *(1989) 169 (4): 1199–1212. https://doi.org/10.1084/jem.169.4.1199; Zangger et al. Pathogens. 2021 Dec; 10(12): 1531. doi: 10.3390/pathogens10121531). Nevertheless, we re-injected $2x10^3$ PFU of the virus for secondary challenge and we know that during acute infection, viral loads can reach up to 10^{5-6} copies/µg in TCRa-/- mice (see Fig 5D) and even 10^{6-7} in CD3-KO mice (see Fig 7-8), although some variations are observed in PCR. This indicates that viral load could have increased upon re-infection if not controlled (as when we injected anti-gd TCR mAb, Fig 5C), which is not the case. This control is consistent with the concomitant increase of γδ T cell response upon re-infection. This is also because of the limit of this successive infection model that we conducted adoptive transfer experiments, and particularly experiments presented in S10 Fig showing that transferred TCM cells persist in uninfected mice (at least 3 weeks)- as it is expected for memory cells - and are still able to protect from lethal infection.*

In addition, we would like to stress that despite persistence of viral load in this model, γδ T cells present a phenotype similar to MCMV-specific inflationary CD8 T cells in wild type mice (Snyder et al. PLoS Pathog 2011 Oct;7(10):e1002295. doi: 10.1371/journal.ppat.1002295)– a well admitted MCMV latency model – and not a typical exhausted phenotype observed on T cells responding to persistent viruses (Chung et al. J Exp Med. 2021 Apr 5; 218(4): e20201730. doi: 10.1084/jem.20201730).

As suggested by reviewer, we quantified infectious virus in 2- 3 months infected TCRa-/- mice by plaque forming unit assay. No infectious virus was detected in liver and lung. In salivary glands (SG), the title varied depending on the mouse:

Mouse 1: $1,5.10^3$/mL (4 mL/SG)
Mouse 2: 2.10^4
Mouse 3: 3.10^5
Mouse 4: $2,5.10^3$
Mouse 5: $3,5.10^3$
Mouse 6: 5.10^2
Mouse 7: 3.10^3
Mouse 8: 8.10^4

Despite the difficulty to compare our results with other studies that don't necessarily use similar mouse strain, infection route, viral strain/origin…, the titer found appears higher than expected in 2 months infected C57BL/6 wild-type mice (Brunel et al. PLoS Pathog 20(1): e1011852. doi.org/10.1371/journal.ppat.1011852 January 18, 2024). So, although γδ T cells were found in SG from MCMV infected mice (Cavanaugh et al. J. Virol., Feb. 2003, p. 1703–1717 Vol. 77, No. 3. DOI: 10.1128/JVI.77.3.1703–1717.2003), they are probably not sufficient to fully control the virus.

As mentioned above we decided to delete Figure 4B, and we modified the text (p18).

Minor issues

1. In Figure 5D, the authors now show that the depletion of gd T cells prior to MCMV primary infection results in no effects on viral titer at dpi 7. Nevertheless, the effects are prominent if the depletion occurs later with a similar waiting time of 7 days. How can this be? The authors need to discuss these data.

*We would like to clarify that the GL3 mAb does not deplete gamma-delta T cells but induces the internalization of γδ TCR (*Koenecke et al, Eur J Immunol. 2009 Feb;39(2):372-9. doi: 10.1002/eji.200838741.*). Hence, these results indeed show that this is not the duration of the treatment by GL3 with is important, but the timing when γδ T cells are treated. The data indicate that at least 2 weeks of priming are required for γδ T cell antiviral function to be active. This now better explained in the text (p. 11)*

Also, the marking next to the y axis in Fig. 5D spells ADN, instead of DNA.

Thanks to reviewer for it attentive reading. We have changed this label.

2. The methods used to perform the cluster analysis in Fig. 6C are rather confusing. More detail and careful wording may be sufficient to address this issue.

We modified the paragraph in the material and methods section as such "To evaluate the bias in the γδ TCR repertoires (Miles et al. Immunology and Cell Biology (2011) 89, 375–387; doi:10.1038/icb.2010.139), we computed a distance matrix of pairwise Levenshtein distances (LD) between CDR3aas, using 'stringdist' R package (M.P.J. van der Loo, 2014, The R Journal 6(1):111-122). When two sequences are similar under the defined threshold, LD>1 (i.e., at most one amino acid difference), they were connected and designated as "cluster". CDR3s with more than one amino acid difference from any other sequences are not connected. The number of CDR3aa making up the cluster represents the size of the cluster. The clustering was between d0 and d7R (d0/d7R) and between d21 and d7R (d21/d7R). The mean size of the clusters was compared between d0/d7R and d21/d7R per mouse".

3. In Fig. S11B, it is not clear what is shown by bubble size. Are these cells from infected vs. uninfected mice? Did they compare the nr. of genes per cluster, or the aggregate gene expression levels of genes belonging to a pathway/cluster? Or something else?

We changed the representation and now show (Fig 9D) selected pathways from the Reactome database, that are significantly enriched for up- and down-regulated genes when comparing C5 and C6 clusters (corresponding to γδ TCM and TEM enriched upon MCMV infection, respectively).
